# The Challenge of Bacterial Infections During Intensive Care Unit Stay After Heart Transplantation

**DOI:** 10.1111/tid.70031

**Published:** 2025-04-20

**Authors:** Rita Minucci, Annalisa De Silvestri, Patrizia Cambieri, Marta Corbella, Carlo Pellegrini, Silvia Roda, Chiara Dezza, Stefano Pelenghi, Raffaele Bruno, Mirko Belliato, Elena Seminari

**Affiliations:** ^1^ Infectious Diseases Unit, Fondazione IRCCS Policlinico San Matteo Pavia Italy; ^2^ SSD Biostatistics and Clinical Trial Center, Fondazione IRCCS Policlinico San Matteo Pavia Italy; ^3^ Microbiology and Virology Unit, Fondazione IRCCS Policlinico San Matteo Pavia Italy; ^4^ Clinical, Surgical, Diagnostic and Pediatric Sciences Department University of Pavia Pavia Italy; ^5^ Cardiac Surgery Unit, Fondazione IRCCS Policlinico San Matteo Pavia Italy; ^6^ Department of SC AR2‐Anesthesia and Cardiothoracic ICU Fondazione IRCCS Policlinico San Matteo Pavia Italy

**Keywords:** antimicrobial prohylaxis, bloodstream infections, heart transplant, infections, pneumonia

## Abstract

**Background:**

Infections occurring in the early post–heart transplant (HT) period heavily contribute to morbidity and mortality. Our goal is to evaluate the incidence of hospital‐acquired pneumonia/ventilator‐associated pneumonia (HAP/VAPs) and/or bloodstream infections (BSIs) after HT during the intensive care unit (ICU) stay and identify their associated risk factors in our tertiary hospital.

**Methods:**

Observational prospective study including all adult patients who consecutively underwent HT from January 1, 2015 to August 31, 2023 at Fondazione IRCCS Policlinico San Matteo, Pavia, Italy. HAP/VAPs and BSIs diagnosed during ICU were included in the analysis.

**Results:**

A total of 106 patients were included, 38 of whom had at least one infectious episode (35.8%), for a total of 57 independent episodes and their incidence was 2.2 per 100 days (95% CI 1.7–2.8). Length of ICU stay was 8 days (IQR: 6–11) for patients without infectious events and 27 days (IQR 14–52) for those with infectious events (*p* < 0.001).

Gram‐negative bacteria were associated with 62.8% of BSIs (mainly *Enterobacterales*) and with 77.9% of HAP/VAP, in this setting *Pseudomonas aeruginosa* accounted for 17.6% of infections while *Klebsiella* spp. accounted for 22.1% of infections.

Colonization with resistant bacteria (HR 2.21, 95% CI 1.12–4.35) was associated with increased risk of infections while perioperative antimicrobial prophylaxis (PAP) covering Gram‐negative bacteria at transplant (HR 0.45, 95% CI 0.23–0.90, *p* = 0.023) was a protective factor.

**Conclusion:**

This study shows that Gram‐negative infections represent the major challenge for HT patients during ICU stay and shows some evidence in support of the PAP covering Gram‐negative infections at transplant.

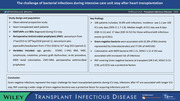

## 1 Introduction

Infections occurring in the early post–heart transplant (HT) period heavily contribute to both morbidity and mortality [1, 2]. In this timeframe, the hospital mortality rate may be as high as 10% and infections associated with PGD continue to be the major causes of unfavorable outcomes [1, 2, 3]. Bacterial infections are the most common type of infection following a HT, occurring in up to 80% of patients, with multidrug‐resistant (MDR) bacteria infections diagnosed in up to 20% of patients during the first 6 months [1, 4]. A prolonged mechanical ventilation, the extracorporeal mechanical oxygenation (ECMO) following HT, the age of the recipient, and a prior cardiac surgery are independent risk factors for early bacterial and fungal infections [4, 5]. A prolonged hospital stay pre–HT is associated with an elevated risk of MDR infections, particularly bloodstream infections (BSIs) and skin soft tissue infections (SSTIs) [1]. Furthermore, it has been proven that a significant risk factor for infections in solid organ transplants (SOT) is represented by rectal colonization with MDR bacteria [4]. European Society of Clinical Microbiology and Infectious Diseases (ESCMID) guidelines suggest a good clinical practice is to perform the screening of all solid organ recipients for MDR before transplant surgery [6].

Our goal is to evaluate the incidence of hospital‐acquired pneumonia/ventilator‐associated pneumonia (HAP/VAPs) and/or BSIs after heart transplantation specifically during the intensive care unit (ICU) stay, analyze the pathogen's epidemiology, and identify their associated risk factors in our tertiary hospital and transplant center [1, 7].

## 2 Methods

All adult patients (aged 18 years and above) who consecutively underwent HT from January 1, 2015 to August 31, 2023 at Fondazione IRCCS Policlinico San Matteo, Pavia (Italy) were included in this observational prospective study. The study was approved by the local ethic committee (Heart Transplant Registry Protocol, number 68992/2018).

HAP/VAPs and BSIs diagnosed during ICU admission in the posttransplant index hospitalization were recorded and microbiologically documented infections were included in the analysis. Only proven HAP/VAPs diagnosed according to ISHLT criteria [8] were included and recorded in an electronic anonymized database. In BSIs, the source of infection was defined as the primary focus of the bacteremia. All microbiologically documented infections were included in the analysis.

Local indications for perioperative antimicrobial prophylaxis (PAP) changed over the study period. ANtimicrobial prophylaxis (AP) included vancomycin from January 1, 2015 to September 30, 2018 (Period 1) and vancomycin plus piperacillin/tazobactam from October 1, 2018 to August 31, 2023 (Period 2), prophylaxis lasted 48 h in both periods.

All patients received at transplant methylprednisolone as induction immunosuppressive therapy (possibly associated with antithymocyte globulins in case of a positive crossmatch), followed by treatment with a chronic maintenance therapy (calcineurin inhibitors, mycophenolate mofetil, and steroids). A preemptive strategy was implemented for the treatment of *Cytomegalovirus* (CMV)‐related disease, consisting of prospective weekly evaluation of the level of whole blood CMV DNA copies. A threshold CMV DNA whole blood level > 10^4^ copies/mL was chosen to start (val)ganciclovir treatment.

### 2.1 Microbiology

Samples from positive blood culture (BC) bottles are subjected to Gram staining and subcultures are performed onto appropriate media: aerobic sheep blood agar plates, selective agar plates, and anaerobic Schaedler agar and 5% sheep blood plates (bioMérieux SA, Marcy‐l'Etoile, France). Species identification and determination of antibiotic susceptibility profiles are carried out by MALDI‐TOF MS (Bruker Daltonics, Bremen, Germany) using the software Bruker Biotyper 3.1 and Phoenix M50 BD (Becton Dickinson, Franklin Lakes, United States) and interpreted according to the 2024 European Committee on Antimicrobial Susceptibility Testing (EUCAST) clinical breakpoints. The presence of carbapenem resistance mechanism is confirmed with the NG‐test Carba 5 immunochromatographic assay (NG Biotech Laboratories). All results both preliminary and final are made available to the clinician in real time h24 7/7, they are communicated by telephone by microbiologist. Coagulase‐negative staphylococci, aerobic and anaerobic diphtheroids, *Micrococcus* spp., *Bacillus* spp., and viridans streptococci are classified as contaminants if only one bottle is positive [9].

Bronchoalveolar lavage (BAL) samples are obtained in all patients with radiological evidence of pneumonia and/or suggestive clinical data. Specimens are firstly subjected to microscopic examination (Gram staining for evaluation of the microbial population and cellularity; detection of extracellular and intracellular bacteria and differential count of macrophages, neutrophils, eosinophils, lymphocytes, bronchial epithelial cells, and squamous epithelial cells). The results of the microscopic examination, if clinically significant, are promptly communicated by telephone to the ward physicians to assess the need for any further rapid molecular investigations (syndromic multiplex panels) that would allow the result of the culture examination to be anticipated by about 12–18 h. The sample is subsequently seeded for culture examination (blood agar, MacConkey agar selective‐differential medium for *Enterobacterales* and Gram‐negative Bacilli not fermenting glucose, chocolate agar, Sabouraud dextrose agar with chloramphenicol and gentamicin) and incubated. The plates are examined 24 and 48 h after incubation, the number of colonies per plate is evaluated and the microbial load expressed in CFU/mL is reported. All pathogenic microorganisms are identified.

The cutoff for pathogenic flora of 104 CFU/mL for BAL also allows infections under antibiotic therapy and initial clinical pictures to be included; in the case of chemo‐antibiotic therapy, however, bacterial loads ≤ 103 are also considered significant.

Species identification and determination of antibiotic susceptibility profiles are carried out by MALDI‐TOF MS (Bruker Daltonics, Bremen, Germany) using the software Bruker Biotyper 3.1 and Phoenix M50 BD (Becton Dickinson, Franklin Lakes, United States) and interpreted according to the 2024 European Committee on Antimicrobial Susceptibility Testing (EUCAST) clinical breakpoints. An active surveillance program to detect rectal colonization has been implemented in our center since January 1, 2014 after ICU admission. Rectal swabs are performed on admission day and weekly thereafter until discharge, for detection of extended‐spectrum beta lactamase (ESBL) *Enterobacterales*, carbapenem‐resistant *Enterobacterales* (CRE), carbapenem‐resistant *Acinetobacter baumannii* (CRAB), and vancomycin resistant *Enterococcus faecium* (VRE). Samples are collected with ESwab REGULAR swabs or Copan in Nylon Flocked Swab liquid medium with 1 mL of modified Amies liquid medium. Screening to identify carriers is done by cultures on chromogenic/selective media or alternatively, for urgent cases, by a nucleic acid amplification (NAAT) molecular test. In the case of positivity on chromogenic/selective media, the colonies are identified at the species level, using identification methods (e.g., MALDI‐ToF mass spectrometry, identification systems based on biochemical profiling, and molecular biology tests). Identification of the type of carbapenemase produced is performed by rapid immunochromatographic method or NAAT assay. In the case of screening positivity to the NAAT molecular test, the surveillance sample should be cultured to isolate and identify the strain and confirm the type of enzyme produced, using the same methods described for isolation from clinical specimen. MDR and ESBL bacteria were defined according to Magiorakos et al. [10].

### 2.2 Statistical Analysis

Categorical variables were described by means of count and percentage, quantitative ones by means of median and Interquartile Range (IQR: 75° percentile minus 25° percentile). Chi‐square test or Fisher's exact test were used in univariate analyses for categorical variables, Mann–Whitney for continuous variables (normality was tested by the Shapiro–Wilk test).

Cox regression hazard model was applied for univariable and multivariable analysis. Two models were fitted, the first model included time to first infection (BSI and/or HAP/VAP) as the dependent variable, the second model was a multi‐failure model considering all infection episodes occurring in a patient as dependent variable. Time of observation starts at transplant and ends at first event or at discharge from ICU in the first analysis or at discharge from ICU in the latter analysis.

Covariates included in the analysis included the following potential clinical and demographic (baseline or time‐dependent) risk factors for infections: age at transplant, gender, extracorporeal membrane oxygenation (ECMO) in place at transplant, left ventricular assist device (L‐VAD), body mass index (BMI) at transplant, total white blood cell count (WBC) at transplant, lymphocyte count at transplant, plasma creatinine value at transplant, PGD after transplant [11], redo procedure (at least one previous open heart surgery for any reason), rectal colonization with ESBL or CRE, CRAB or VRE at transplant; zenit CMV‐DNA on whole blood during ICU stay (categorized as undetectable, positive up to 9999 copies/mL, positive > 10 000 copies/mL, and < 100 000 and positive > 100 000 copies/mL), and type of antimicrobial prophylaxis at transplant.

Non collinear covariates clinically important or with *p* < 0.20 at univariate models were included in multivariate ones following a stepwise procedure. Results are expressed as hazard ratio (HR) with 95% confidence interval (95% CI). A *p* value < 0.05 is considered significant; all analyses are performed with Stata v 18.0 (Statacorp USA).

## 3 Results

### 3.1 Demographic and Patients Characteristics

A total of 106 patients were included in the present study. Patients' characteristics are summarized in Table 1. Total 72 of patients (68%) were males with a median age of 57 y/o (1–3 IQR 46–59). L‐VAD as bridge to transplant was implanted in 7 (6.6%) patients while ECMO in 13 (12.2%), 8 patients in ECMO were on renal replacement therapy at transplant; PGD was diagnosed in 16 (15.09%) patients. Patients at high risk for CMV infection (defined as positive donor CMV serology and negative recipient CMV serology) were 8 out of 106 (7.5%). Five patients (4.7%) had an acute rejection during ICU stay requiring steroids boluses.

**TABLE 1:** Patient's characteristics.

Patients (106)	
Gender	
Male	72 (68%)
Female	34 (32%)
Age (years)	57.1 (46.42–59.39)
BMI	23.9 (21.6–26.8)
WBC (cell/uL)	8120 (6600–9850)
Lymphocites (cell/uL)	1500 (890–1378)
Creatinine (mg/dL)	1.19 (0.89–1.37)
REDO procedure	42 (39.6%)
L‐VAD pretransplant	7 (6.6%)
ECMO at transplant	13 (12.2%)
ICU stay (days)	13 (7–24)
MDR rectal colonization	27 (25.47%)
Primary graft disease	16 (15.1%)

*Note*: Continuous data reported as median (1–3 IQR).

Four patients were treated with antibiotic therapy at transplant for a concomitant infections (3 patients with L‐VAD driveline infection and 1 patient with BSI due to VRE on treatment at transplant).

Antimicrobial prophylaxis included vancomycin in 53 patients in Period 1 and vancomycin plus piperacillin tazobactam in 48 patients in Period 2 (five severely ill patients colonized with MDR bacteria received a targeted antimicrobial prophylaxis as deemed at high risk for subsequent infection).

### 3.2 Microbiology

The pathogens associated with infections are summarized in Table 2. Gram‐negative bacteria were associated with 62.8% of BSIs (mainly represented by *Enterobacterales*). Carbapenem resistant strains accounted for 8.6% of BSI by Gram‐negative bacteria, while ESBL for 9.1%. Gram‐positive bacteria accounted for 30.2% of infections, mainly represented by methicillin resistant *Staphylococcus aureus* or *Staphylococcus epidermidis*.

**TABLE 2:** Microbiologic isolates.

BSI (38)			Pneumoniae (68)		
**Gram negative**	**22**	**57.9%**	**Gram negative**	**53**	77.9%
*K. pneumoniae*	3	7.9%	*K.pneumoniae*	5	7.4%
*K. pneumoniae KPC*	4	10.5%	*K. Pneumoniae KPC*	4	5.9%
*K. oxytoca*	2	5.2%	*K. pneumoniae ESBL*	1	1.5%
*K. oxytoca ESBL*	1	2.6%	*K. oxytoca*	5	7.4%
*E. coli*	2	5.2%	*E.coli*	7	10.3%
*S. marcescens*	1	2.6%	*Citrobacter koseri*	2	2.9%
*S. marcescens ESBL*	1	2.6%	*S. marscenscens*	5	7.3%
*Hafnia alvei*	1	2.6%	*H. influenzae*	2	2.9%
*E. cloacae*	1	2.6%	*P. mirabilis*	2	2.9%
*H. influenzae*	1	2.6%	*E. aerogenes*	1	1.5%
*P. mirabilis*	1	2.6%	*E. cloacae VRE*	1	1.5%
*F. nucleatum*	1	2.6%	*A. baumanii*	2	2.9%
*S. maltophilia*	1	2.6%	*S. maltophilia*	3	4.4%
*P. aeruginosa*	1	2.6%	*B. gladioli*	1	1.5%
*P. aeruginosa ESBL*	1	2.6%	*P. aeruginosa*	9	13.2%
			*P. aeruginosa VIM*	3	4.4%
**Gram positive**	**11**	28.9%	**Gram positive**	8	11.8%
*S. aureus (MRSA)*	2	5.2%	*E. faecium*	2	2.9%
*S. epidermidis (MRSE)*	5	13.2%	*E. faecium VRE*	1	1.5%
*S. oralis*	1	2. 6%	*Corynebacterium estriatum*	1	1.5%
*G. adiacans*	1	2.6%	*S. aureus MRSA*	2	2.94%
*E. faecium (VRE)*	2	5.2%	*S. aureus MSSA*	2	2.94%
**Fungi**	**5**	13.2%	**Fungi**	**7**	10.29%
*C. parapsilosis*	4	10.5%	*A. flavus*	2	2.94%
*C. albicans*	1	2.6%	*A. terreus*	1	1.47%
			*A. niger*	1	1.47%
			*A. fumigatus*	2	2.94%
			*Geotrichum*	1	1.47%

The same picture has been observed for HAP/VAP associated isolates, 77.9% of infections were attributable to Gram‐negative pathogens, *Pseudomonas aeruginosa* accounted for 17.6% (4.4% were carbapenemase‐resistant) of infections while *Klebsiella* spp. accounted for 22.1% (5.8% were carbapenemase‐resistant) of infections. HAP/VAP was the source of infection in 39.4% of BSIs (the same pathogen was isolated in BAL and BCs). *Aspergillus* spp. was isolated in BAL culture in 6 patients, all were classified as probable invasive pulmonary aspergillosis according to ISHLT criteria [8, 12].

All the patients underwent screening for colonization at the time of transplantation, of these 14 (14.8%) were identified as colonized. Posttransplant, during ICU stay, all the patients repeated the rectal swabs weekly, and 13 (12.3%) patients resulted to be colonized during their ICU stay (1 patient was colonized by 2 different bacterial species). In the end, 27 (25.5%) patients resulted to be colonized. Microbiological isolates identified in rectal swabs included *K. pneumoniae* KPC (11 cases), vancomycin resistant *E. faecium* (8 cases), and ESBL *Enterobacteriaceae* (10 cases). Rectal colonization at transplant was more frequent in Period 2 compared with Period 1 (4.3% vs. 21.1%, *p* = 0.012), and the trend was consistent also for colonization starting during ICU stay (10.4% vs. 22.8%, *p* = 0.09). Among colonized patients, 11 (40.7%) patients were diagnosed with BSI and VAP/HAP due to the same pathogen.

### 3.3 Infectious Episodes

A total of 38 patients had at least one infectious episode (35.8%), for a total of 57 independent episodes and their incidence was 2.2 per 100 days (95% CI 1.7–2.8). Length of ICU stay was 8 days (IQR: 6–11) for patients without infectious events and 27 days (IQR 14–52) for those with infectious events (*p* < 0.001).

Median time to the first infectious event was 5 days (IQR: 3–9). Colonization with MDR bacteria (HR 2.21, 95% CI 1.12–4‐35) was associated with increased risk of infections while PAP covering Gram‐negative bacteria at transplant (HR 0.45, 95% CI 0.23–0.90, *p* = 0.023) and a CMV DNA plasma value below 10^4^copies/mL (HR 0.38, 95% CI 0.16–0.90, *p* = 0.028) were protective factors (Table 3).

**TABLE 3:** Univariable and multivariable analysis.

	First infection univariate analysis (95% CI)	*p*	First infection multivariate analysis (95% CI)	*p*	All infections univariate analysis (95% CI)	*p*	All infections multivariate analysis (95% CI)	*p*
Gender	1.5 (0.70–3.23)	0.29	1.14 (0.46–2.80)	0.77	0.15 (0.8–2.78)	0.17	1.05 (0.5–2.2)	0.90
MDR rectal colonization	2.02 (1.02–3.94)	0.04	3.06 (1.24–7.58)	0.02	1.6 (0.97–2.6)	0.06	2.31 (1.17–4.56)	0.02
Prophylaxis at transplant	0.48 (0.25–0.93	0.03	0.4 (0.16–0.99)	0.05	0.55 (0.34–0.88)	0.01	0.48 (0.24–0.96)	0.04
Primary graft disease	1.77 (0.87–3.61)	0.12	1.33 (0.47–0.71)	0.59	1.64 (0.97–2.76)	0.60	1.85 (0.88–3.88)	0.1
ECMO at transplant	1.83 (0.87–3.82)	0.11			1.7 (1.01–2.91)	0.045		
REDO procedure	1.89 (0.96–3.72)	0.07	1.00 (0.37–2.74)	0.99	1.78 (1.07–2.97)	0.03	1.38 (0.62–3.10)	0.43
L‐VAD pretransplant	0.39 (0.05–3.10)	0.38			0.46 (0.14–1.56)	0.21		
Age	1.02 (0.98–1.1)	0.30			1.00 (0.98–1.03)	0.43		
WBC	1.03 (0.97–1.09)	0.39	1.0 (0.93–1.09)	0.93	1.04 (0.98–1.08)	0.13	1.00 (0.94–1.07)	0.98
Lymphocyte	0.97 (0.69–1.37)	0.88			0.88 (0.66–1.17)	0.37		
Creatinine	1.39 (0.79–2.44)	0.26	1.94 (0.74–5.04)	0.20	1.24 (0.82–1.87)	0.30	1.33 (0.75–2.36)	0.33
BMI	1.05 (0.96–1.14)	0.32			1.03 (0.96–1.10)	0.37		
CMV‐DNA (copies/mL)								
1–9999	0.85 (0.38–1.92)	0.7	0.45 (0.16–1.29)	0.14	0.78 (0.40–1.51)	0.47	0.37 (0.15–0.88)	0.03
10 000–100 000	1.18 (0.47–2.98)	0.72	1.22 (0.4–3.72)	0.73	1.34 (0.71–2.52)	0.36	1.40 (0.60–3.31)	0.44
> 100 000	1.28 (0.42–3.97)	0.66	1.30 (0.33–5.18)	0.71	1.58 (0.76–3.30)	0.22	1.92 (0.70–5.22)	0.20

While considering only the first infectious episode, being colonized with a resistant strain at transplant was associated with increased risk of having at least one infectious episode during ICU stay (HR 3.1, 95% CI 1.2–7.6, *p* = 0.02), PAP with Gram‐negative coverage at transplant was protective (HR 0.4, 95% CI 0.16–0.99, *p* = 0.049) (Table 3).

### 3.4 BSIs

A total of 35 BSIs were diagnosed during the study period in 24 patients, three of these were polymicrobial infections (8.6%). The BSI rate was 0.9 per 100 patient/ICU days (95% CI 0.6–1.3). Median time to BSI occurrence was 9 days (IQR: 3–18). Antimicrobial prophylaxis at transplant was a protective factor (HR 0.42, 95% CI 0.18–0.99 *p* = 0.047).

### 3.5 HAP/VAP

A total of 32 pneumonia episodes were diagnosed during the study period in 31 patients and the pneumonia rate was 1.7 per 100 patient/ICU days (95% CI 1.3–2.3). Median time to HAP/VAP occurrence was 4 days (IQR 3–7). None of the variables analyzed were either a risk or a protective factor for HAP/VAP in ICU.

### 3.6 Mortality

Total 15 (14.2%) patients died in ICU during the study period. Death was associated with PGD (HR 58.8, 95% CI 6.9–500, *p* < 0.0001) and increased creatinine value at transplant (HR 5.2, 95% CI 1.01–26.6, *p* = 0.048). Four deaths were associated with a Gram‐negative BSI episode associated with septic shock, all events occurred in Period 1.

## 4 Discussion

In our study, we found that bacterial infections (both BSIs and/or HAP/VAP) occurred in 35.8% of patients admitted in ICU following HT. Data presented define a marked increase in prevalence of infections if compared to the approximately 20% infection rates reported in previous decades [13, 14, 15, 16] and are more in agreement with epidemiology of recent years [17, 18, 19]. The majority of infections were sustained by Gram‐negative bacteria, responsible for 62.8% of BSI and 77.9% of HAP/VAP. In our experience, as in others, *Klebsiella* spp. and *P. aeruginosa* represent the bacteria most frequently causing BSIs and HAP/VAP in heart transplanted patients [18]. The source of infection for BSIs in this subset is often represented by concomitant pneumonia [20] as in the present study where pneumonia sustained by the same pathogen was recorded in 39.4% of BSIs. Bacteremic pneumonia has not been associated with mortality in critically ill immunocompromised patients with acute hypoxic respiratory failure, while comorbidities at baseline (as documented by Charlson index) were [20]. Nonetheless, penetration of antibiotics into pulmonary parenchyma might be reduced compared with plasma levels, so optimization of their dosage regimens in HAP/VAP may be required [21].

Interestingly, 31.4% of the MDR colonized patients experienced one or more infectious events caused by the same germ. The reported incidence for infection after colonization is in median 6.5 days [22]. Colonization could represent a significant risk factor for the onset of infectious events in the early posttransplant period [23] and our results confirmed that being colonized by a resistant strain at transplant or in the following time period was independently associated with increased risk of having at least one infectious episode during the ICU stay [22].

Rectal colonization at the time of transplantation was significantly more frequent in Period 2 compared to Period 1 and this trend was also observed in patients who became colonized later during ICU stay. The increase of rectal colonization by MDR organisms could be explained by changes in hospital epidemiology over the years, especially following the COVID‐19 pandemic [24, 25]. Other factors such as prolonged duration of ICU stays and bridging procedures, including ECMO or L‐VAD, prior to transplantation likely contribute to this trend [1]. The advance of the surgical techniques, the new allocation policies and the improvement of supportive techniques and therapeutics in patients with end‐stage organ disease have enhanced access to the organ transplantation, but at the same time these progresses have elevated the risk of acquired MDR colonization before and after transplant [23]. Our data support the use of screening procedures for MDR colonization during the posttransplant period, as MDR strains can be acquired posttransplant, for both epidemiological and clinical reasons [6, 26–28].

Considering the rate of infections due to Gram‐negative bacteria in the posttransplant period, the indications for PAP at transplant changed in our tertiary center in 2018. From October 2018, PAP with vancomycin plus a coverage anti Gram‐negative (piperacillin/tazobactam) was implemented. Despite not being formally foreseen by guidelines, that suggest the use of a glycopeptide and a first‐generation cephalosporin [29, 30], a PAP covering a wider range of Gram‐negative bacteria is applied in roughly 30% of centers worldwide [31, 32], considering the high rate of surgical site infections sustained by Gram‐negative bacteria after cardiac surgery [29, 30, 31, 32, 33]. The benefit of this strategy in reducing the incidence of infectious episodes should be balanced to avoid the widespread use of unnecessary antibiotics. In the present study, the use of a PAP comprehending Gram‐negative coverage was associated with a reduction in infection incidence in the posttransplant period, mainly BSIs. The pros associated with anti Gram‐negative coverage include that patients without infections were hospitalized in the ICU for a shorter period of time than those who were, the majority of Gram‐negative bacteria responsible for infections remained susceptible to beta lactams antibiotics and PAP has been administered for a short period of administration time. The cons of this strategy is the potential selection of resistant bacteria. In an era of antimicrobial stewardship programs required by the spread of multidrug resistant bacteria, we agree that the strategy of covering Gram‐negative bacteria in HT should be supported with data prospective clinical trials, though they are not easily performed.

A recent Cochrane meta‐analysis on perioperative antibiotics prophylaxis in SOT recipients underlines the uncertainty in field, in the absence of proper conducted randomized trials [34]. The discussion about this topic is open, and recently the ESCMID/EUCIC clinical practice guidelines suggests to consider the use of targeted PAP for all SOT recipients who are colonized with ESBL before surgery [6]. Certainly, in the future, the recognition of those patients at major risk of postsurgical infectious complications through predictive models or algorithms for risk assessment will help physician in prescribing the better PAP tailored based each patient's characteristics [35].

CMV replication was associated with an increased risk of bacterial infection due to its immunomodulatory nature and, in particular, with an increased risk of mortality in SOT recipients with carbapenemase producing *Enterobacterales* BSI [36]. In addition, active CMV replication is considered a surrogate marker of the net‐state of immunosuppression in the early posttransplant period and this could explain the increased risk of infection observed [36, 37]. In our study, we found a CMV DNA plasma value below 10^4^ copies/mL is associated with a lower risk of bacterial infection, consistent with what is reported in literature. Differently from the literature, the total WBC count and lymphocyte count at the time of HT is not correlated with an increased risk of infection, as instead reported in case of liver transplantation [38]. An association between lower absolute lymphocyte count levels and increased rates of sepsis‐related mortality, as well as a higher incidence of bacteremia has been observed [38]. It is proven that lymphocyte count is a marker of nutritional status and humoral and cellular immune response. In addition, since lymphocyte count is influenced by nutritional status, it is evident that malnutrition weakens the host immune system, raising the risk of infections or other complications [38, 39]. A possible explanation for lack of association between lymphopenia and higher risk of infection in heart transplanted patients is that compared with patients with end‐stage liver disease, patients listed for heart transplantation normally have lymphocytes counts within normal range. The same has been observed in our cohort for BMI value at transplant, that was not associated with increased risk of infections, possibly because observed median BMI was within normal range. Considering nutritional state is crucial, a low BMI was linked to a higher risk of infection complications after transplant [40]. A retrospective analysis of the International Society for Heart and Lung Transplantation (ISHLT) Registry revealed that an underweight pre–HT BMI is associated with a greater risk of death due to infection posttransplant [41]. In our study, we find a significant association between mortality and primary graft disease, as well as an increase in creatinine value at the time of transplant. Although significant advances have been made in post–HT, PGD remains one of the leading cause of early mortality after HT. It's defined as a severe left, right, or biventricular dysfunction within 24 h following the transplantation, in absence of a secondary cause [10, 42–44].

It has an incidence of 20%–30%, that may continue to increase due to new allocation policies (such as the increase in age of both donors and recipients or utilization of high‐risk donors) [42, 45].

The occurrence of infectious events (BSI and HAP/VAP) was not linked to mortality, and death resulting from Gram‐negative BSI only happened in the initial period, as previously described [18]. It's important to note that the use of newer drugs for MDR infections (β‐lactam–β‐lactamase inhibitor combinations) have dramatically reduced the mortality associated with MDR bacterial infection [46, 47, 48, 49], as well the shorter time to effective antimicrobial treatment therapy among patients carrying MDR strains [50].

The limitations of the present study are mainly represented by being a monocenter study so representing an institution‐specific patient population, moreover it covers a wide time range in which microbiology ecosystem has changed in our hospital (also following Covid 19 epidemic), in particular rectal colonization was more frequent in Period 2 compared with Period 1. In this timeframe new drugs have become available to treat Gram‐negative MDR infections, such as new β‐lactam–β‐lactamase inhibitor combinations that have changed the prognosis of infections due to MDR bacteria, for this reason despite a higher number of rectal colonized patients observed in Period 2, no deaths occurred due to the same pathogen. In addition, we do not have information regarding MDR bacterial whole genome sequencing for epidemiological monitoring of clinical isolates for a better evaluation of colonized and infected patients.

In summary, this study shows that Gram‐negative infections represent the major challenge for heart transplanted patients during ICU stay, moreover infections after HT are associated with longer ICU stay, despite mortality was comparable among patients with and without infections. PAP covering a wider range of Gram‐negative bacteria was a protective factor for acquiring infections post–HT.

## Author Contributions

**Rita Minucci**: data curation, investigation, writing – review and editing. **Annalisa De Silvestri**: conceptualization, writing – original draft, software, formal analysis. **Patrizia Cambieri**: conceptualization, investigation, validation, data curation. **Marta Corbella**: writing – original draft, visualization, project administration. **Carlo Pellegrini**: writing – review and editing, supervision. **Silvia Roda**: conceptualization, investigation, writing – original draft, data curation, supervision. **Chiara Dezza**: conceptualization, visualization, resources. **Stefano Pelenghi**: investigation, funding acquisition, visualization, writing – review and editing. **Raffaele Bruno**: writing – review and editing, project administration, resources, supervision. **Mirko Belliato**: investigation, writing – original draft, data curation. **Elena Seminari**: conceptualization, investigation, funding acquisition, writing – original draft, methodology, project administration, resources.

## Supporting information


Supporting Information
