# Supplementary material for: The Challenge of Bacterial Infections During Intensive Care Unit Stay After Heart Transplantation
Source: Transpl Infect Dis. 2025 Apr 20;27(3):e70031. doi: 10.1111/tid.70031 (PMC12205279; doi:10.1111/tid.70031)
Supplement: Supplementary file 1 — Supporting Information [file TID-27-e70031-s001.pptx]

## Slide 1
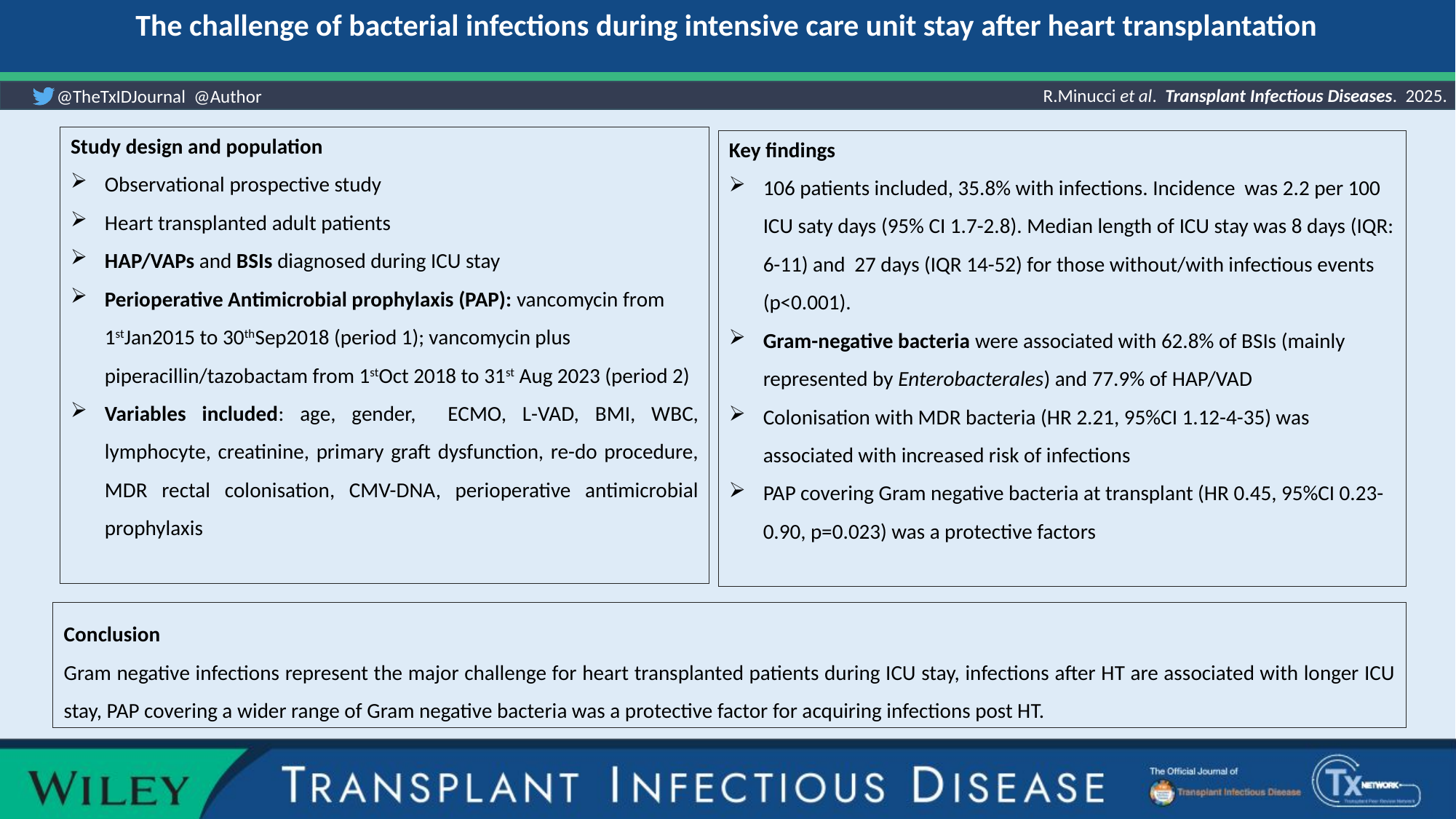

The challenge of bacterial infections during intensive care unit stay after heart transplantation
R.Minucci et al. Transplant Infectious Diseases. 2025.
 @TheTxIDJournal @Author
Study design and population
Observational prospective study
Heart transplanted adult patients
HAP/VAPs and BSIs diagnosed during ICU stay
Perioperative Antimicrobial prophylaxis (PAP): vancomycin from 1stJan2015 to 30thSep2018 (period 1); vancomycin plus piperacillin/tazobactam from 1stOct 2018 to 31st Aug 2023 (period 2)
Variables included: age, gender, ECMO, L-VAD, BMI, WBC, lymphocyte, creatinine, primary graft dysfunction, re-do procedure, MDR rectal colonisation, CMV-DNA, perioperative antimicrobial prophylaxis
Key findings
106 patients included, 35.8% with infections. Incidence was 2.2 per 100 ICU saty days (95% CI 1.7-2.8). Median length of ICU stay was 8 days (IQR: 6-11) and 27 days (IQR 14-52) for those without/with infectious events (p<0.001).
Gram-negative bacteria were associated with 62.8% of BSIs (mainly represented by Enterobacterales) and 77.9% of HAP/VAD
Colonisation with MDR bacteria (HR 2.21, 95%CI 1.12-4-35) was associated with increased risk of infections
PAP covering Gram negative bacteria at transplant (HR 0.45, 95%CI 0.23-0.90, p=0.023) was a protective factors
Conclusion
Gram negative infections represent the major challenge for heart transplanted patients during ICU stay, infections after HT are associated with longer ICU stay, PAP covering a wider range of Gram negative bacteria was a protective factor for acquiring infections post HT.
